# Sustainable resistance switching performance from composite-type ReRAM device based on carbon Nanotube@Titania core–shell wires

**DOI:** 10.1038/s41598-020-75944-3

**Published:** 2020-11-02

**Authors:** Youngjin Kim, Minsung Kim, Ji Hyeon Hwang, Tae Whan Kim, Sang-Soo Lee, Woojin Jeon

**Affiliations:** 1 The Research Institute of Industrial Science, Hanyang University, Seoul, 04763 Republic of Korea; 2 Department of Electronic and Computer Engineering, Hanyang University, Seoul, 04763 Republic of Korea; 3 Soft Hybrid Materials Research Center, Korea Institute of Science and Technology, Seoul, 02792 Korea; 4 Department of Chemical and Biological Engineering, Korea University, Seoul, 02841 Korea; 5 Department of Advanced Materials Engineering for Information and Electronics, Kyung Hee University, Yongin, 17104 Korea

**Keywords:** Chemistry, Materials science, Nanoscience and technology

## Abstract

A novel nanocomposite-based non-volatile resistance switching random access memory device introducing single-walled carbon nanotube (SWCNT)@TiO_2_ core–shell wires was proposed for flexible electronics. The SWCNT was de-bundled by ultrasonication with sodium dodecylbenzene sulfonate (SDBS), and then the TiO_2_ skin layer on the SWCNT surface was successfully introduced by adding benzyl alcohol as a weak surfactant. The nanocomposite resistance switching layer was composed of the SWCNT@TiO_2_ core–shell wires and poly(vinyl alcohol) (PVA) matrix by a simple spin-coating method. The device exhibited reproducible resistance switching performance with a remarkably narrow distribution of operating parameters (V_SET_ and V_RESET_ were 2.63 ± 0.16 and 0.95 ± 0.11 V, respectively) with a large R_ON_/R_OFF_ ratio of 10^5^ for 200 consecutive switching cycles. Furthermore, the excellent resistance switching behavior in our device was maintained against mechanical stress up to 10^5^ bending test. We believe that the nanocomposite memory device with SWCNT@TiO_2_ core–shell wires would be a critical asset to realize practical application for a flexible non-volatile memory field.

## Introduction

Increasing data storage, which demands computing and imaging electronics, has been necessitating the development of high-performance memory devices with fast operation speed, high data storage, and low power consumption^1–4^. Moreover, ubiquitous platforms with flexible computing systems have become a critical asset for futuristic technology^5,6^. Therefore, it is crucial to achieve excellent and sustainable electrical and mechanical performances in the field of storing information^7^ Among several next-generation memory candidates, resistance switching random access memory (ReRAM) device, which is based on resistance switching phenomenon by two distinguishable resistance states, has attracted much attention due to high operation speed, non-volatility, simple structure, low-power consumption, and compatibility to conventional Si-based fabrication process^1,2,8–10^.

The flexibility has been aroused as a new requirement for the next-generation semiconductor devices due to demonstrating the wearable application. For a flexible application of the ReRAM device, the transition metal oxide (TMO)-based ReRAM devices are widely used due to their excellent resistance switching performance. However, the TMOs have a fatal weakness in flexible applications due to their lack of flexibility. Organic-based ReRAM devices, in contrast, have good flexibility but low yields and poor switching performance. The nanocomposite structures composed of a combination between inorganic clusters as a resistance switchable filler (RSF) and a polymer/organic matrix have currently emerged as the promising components for next-generation flexible ReRAM memory devices because the nanocomposite system can ideally take excellent resistance switching properties of the inorganic material and good mechanical and optical properties of the organic-based material^11–15^.

Carbon-based materials such as graphenes, graphite nanosheets, carbon nanofibers, and carbon nanotube (CNT) have recently attracted much attention owing to their fascinating properties such as a tunable bandgap, high electron mobility, and quantum electronic transport^16,17^. Among them, CNT is a promising material for the flexible electronics due to superior electrical and mechanical properties. Moreover, a large aspect ratio of the CNT has an advantage of electrical percolation to connect between the bottom and the top electrodes of the ReRAM device. *Hwang *et al*.* reported the nanocomposite-based ReRAM device introducing B- and N-doped CNTs dispersed in a polystyrene matrix^18^. *Chaudhary *et al*.* also reported the non-volatile resistive switching effect by a poly(3-hexylthiophene)-CNT composite films^19^. *Zhao *et al*.* reported rewritable composite-type memory based on polymethyl methacrylate-CNT layer^20^, and polyurethane-CNTs composite layer was introduced as a resistive layer by Li group^21^. Although the resistive devices based on charge trap- or filamentary-controlled switching mechanism exhibited notable resistive switching performance with good mechanical flexibility, the nanocomposite-based ReRAM device has a potential problem which is reproducibility in cell-to-cell and device-to-device because randomly distributed and aggregated fillers can still be observed in the matrix. Moreover, the developed functionality on the surface of the CNT was not uniform. Therefore, it is necessary to develop a resistive switchable composite system without relying on the random distribution of the RSFs.

In this paper, we proposed a core–shell structured resistance switchable filler based on single-walled CNT (SWCNT) for flexible ReRAM devices with high resistive switchable performance. The TiO_2_ resistive skin layer to induce resistive switching behavior was uniformly introduced on the surface of the physically de-bundled SWCNTs by weak-surfactant treatment of benzyl alcohol (BA). The nanocomposite resistance switchable layer was prepared by mixing SWCNT@TiO_2_ core–shell wires (ST-CSWs) and poly(vinyl alcohol) (PVA) polymer matrix. Then, resistance switching behavior of the nanocomposite ReRAM device, including ST-CSWs, was evaluated for reproducibility and flexibility. The switching mechanism in the device was investigated by morphological and structural analyses.

## Results and discussion

**Figure 1:**
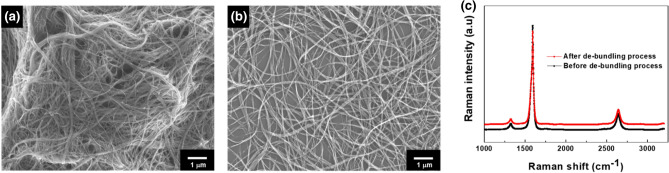
(**a**) SEM images of the SWCNTs (**a**) before and (**b**) after the physically de-bundling process. (**c**) Raman spectra of the SWCNTs before and after the de-bundling process.

The de-bundling process of SWCNTs is essential due to its π-π interaction and van der Waals interaction between them^22^. Chemical treatment with strong acids, such as HNO_3_ or H_2_SO_4_, is widely used for the de-bundling of the CNTs. The strong oxidizing agents preferentially disrupt the aromatic ring structure of the CNTs^23^. Some papers have reported that the disrupted structure of the carbon-based materials can be changed by externally applied bias, being able to induce resistive switching phenomenon^24–27^. In our system, we should avoid the resistive change of the CNT because our concept achieved the resistive switching behavior by employing the TiO_2_ layer on the SWCNT surface. The role of SWCNT is to transport the injected electrons from one electrode to another when the conducting filament is formed in the TiO_2_ resistive switchable layer. Therefore, the de-bundling process with the physical method was employed instead of conventional chemical treatment with strong acid. As shown in Fig. 1a and b, we confirmed that SWCNTs were successfully de-bundled by the physical de-bundling route. The quality of the resulting SWCNTs could be confirmed by the Raman spectroscopy, as shown in Fig. 1c. Raman spectroscopy is an effective method to characterize the bonding detail of carbon-based materials, such as the bonding type, domain size, and internal stress. In the broad Raman peaks, D-band (1322 cm^−1^) is associated with the sp^3^ bonding structure, and G-band (1590 cm^−1^) indicates the sp^2^ bonding structure of the resulting SWCNTs. When the ratio of the intensity of the D and G bands (*I*_*D*_/*I*_*G*_) before and after the physical separation process was calculated, the *I*_*D*_/*I*_*G*_ ratio of resulting SWCNTs before and after the de-bundling process is hardly changed from 0.07 to 0.11, indicating that the resulting SWCNTs were successfully separated without developing defect on its surface^23^.

**Figure 2:**
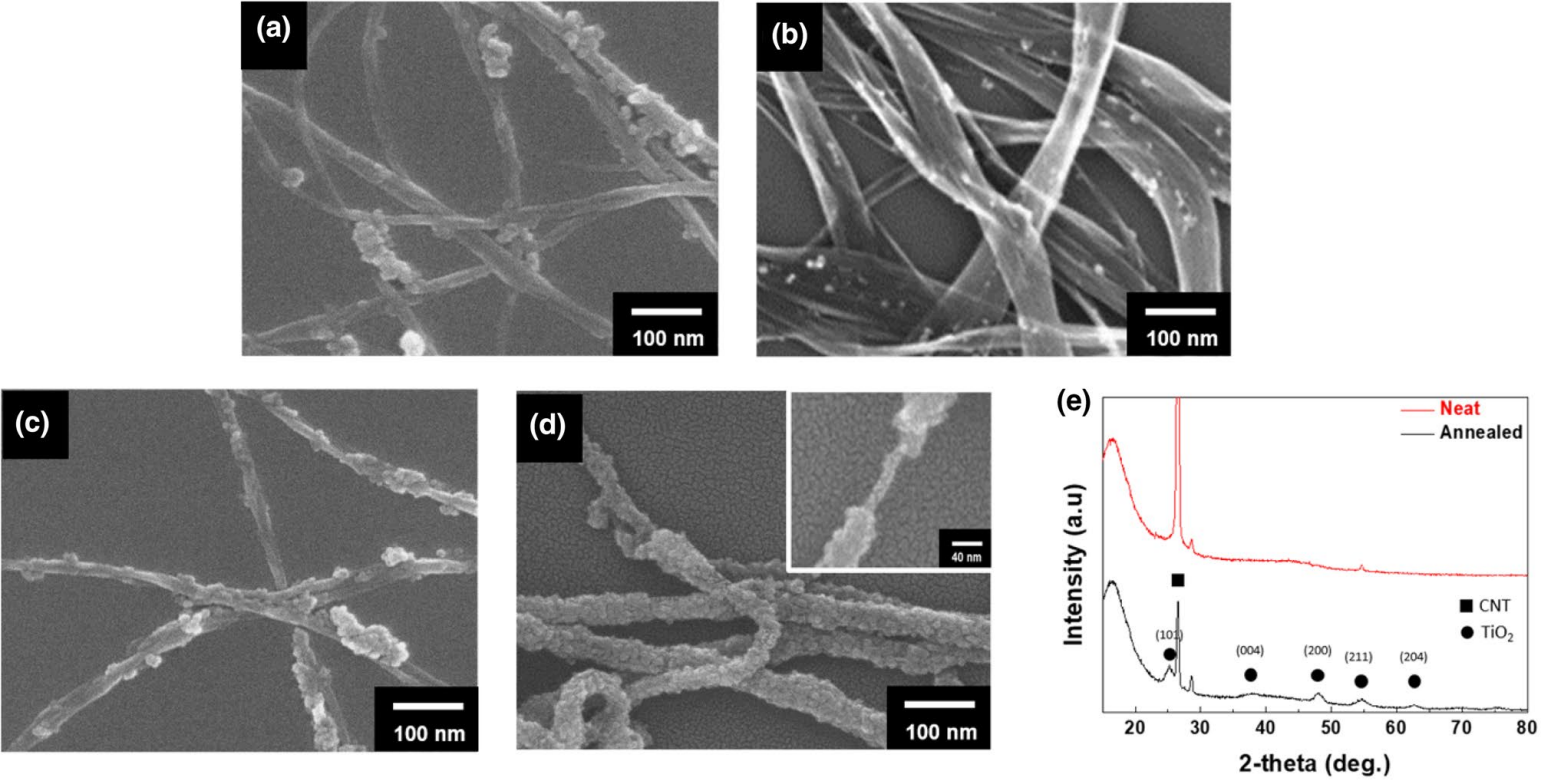
SEM images of the resulting SWCNT@ TiO_2_ core–shell particles (**a**) based on chemically-treated SWCNTs and (**b**) based on physical-treated SWCNTs without BA treatment. SEM images of the SWCNT@ TiO_2_ core–shell particles based on physically-treated SWCNTs with different molar ratios BA of (**c**) m_BA,10_ and (**d**) m_BA,30_. (**e**) XRD spectrum of the ST-CSW before and after the heat treatment at 500 °C.

To introduce the TiO_2_ resistive layer on the SWCNT, the surface characteristics of the SWCNT must be changed because the inherent property of the SWCNT is hydrophobic^28^. When the TiO_2_ is synthesized by the sol–gel method, it is hard to adsorb the TiO_2_ sol on the SWCNT surface^29^. Therefore, the SWCNT surface should be functionalized to provide an adsorption site with the TiO_2_ sol. The common approach is to develop covalent bonding on the SWCNT surface by ultrasonication in strong acid. However, already mentioned above, we have to avoid the destruction of the aromatic ring of SWCNT. Moreover, the acid treatment allows little control over the location, nature, and quantity of the introduced functional groups, leading to a non-uniform coating of the TiO_2_ layer on the SWCNT surface, as shown in Fig. 2a. Thus, BA is considered for introducing a high-quality TiO_2_ layer on the SWCNT surface in this study.

We investigated the effect of BA in the TiO_2_ coating on the SWCNT surface. The molar ratio of BA for a reaction mixture was controlled under the condition of fixing the CNT concentration of 10 wt% of the total weight. The molar ratio of BA (m_BA_) was Ti precursor:BA:EtOH:H_2_O = 0.25:x:30:5, where x was varied with 10 (m_BA.10_) and 30 (m_BA,30_). In the morphological analysis by scanning electron microscopy (SEM) measurement, we could confirm that the introducing BA strongly affects the quality of introducing the TiO_2_ layer on the surface of SWCNTs (Fig. 2c and d), compared with the resulting particles prepared without introducing BA (Fig. 2b). In addition, it was observed that the resulting SWCNTs without BA treatment were reaggregated after the sol–gel reaction. It is because BA is adsorbed on the SWCNT surface by π–π interactions between the aromatic SWCNT surface and the benzyl ring of BA^29,30^. Moreover, the hydroxyl group of the BA provides hydrophilic property on the SWCNT surface, leading the uniform hydrolysis with the titanium precursor^31^. The amount of BA in the reaction mixture also affects the quality of the TiO_2_ coating significantly. According to the increasing molar ratio of BA in the mixture, the TiO_2_ layer tended to coat uniformly on the entire surface of SWCNTs. It is because the high concentration of BA can provide the functionalized sites on the entire surface of SWCNTs. The thickness of the TiO_2_ layer on SWCNT revealed about 15 nm, as shown in the inset image of Fig. 2d. Finally, as shown in Fig. 2e, while the ST-CSW before heat treatment was mostly amorphous nature, the TiO_2_ layer exhibited the crystal structure of the anatase phase after heat treatment at 500 °C.

**Figure 3:**
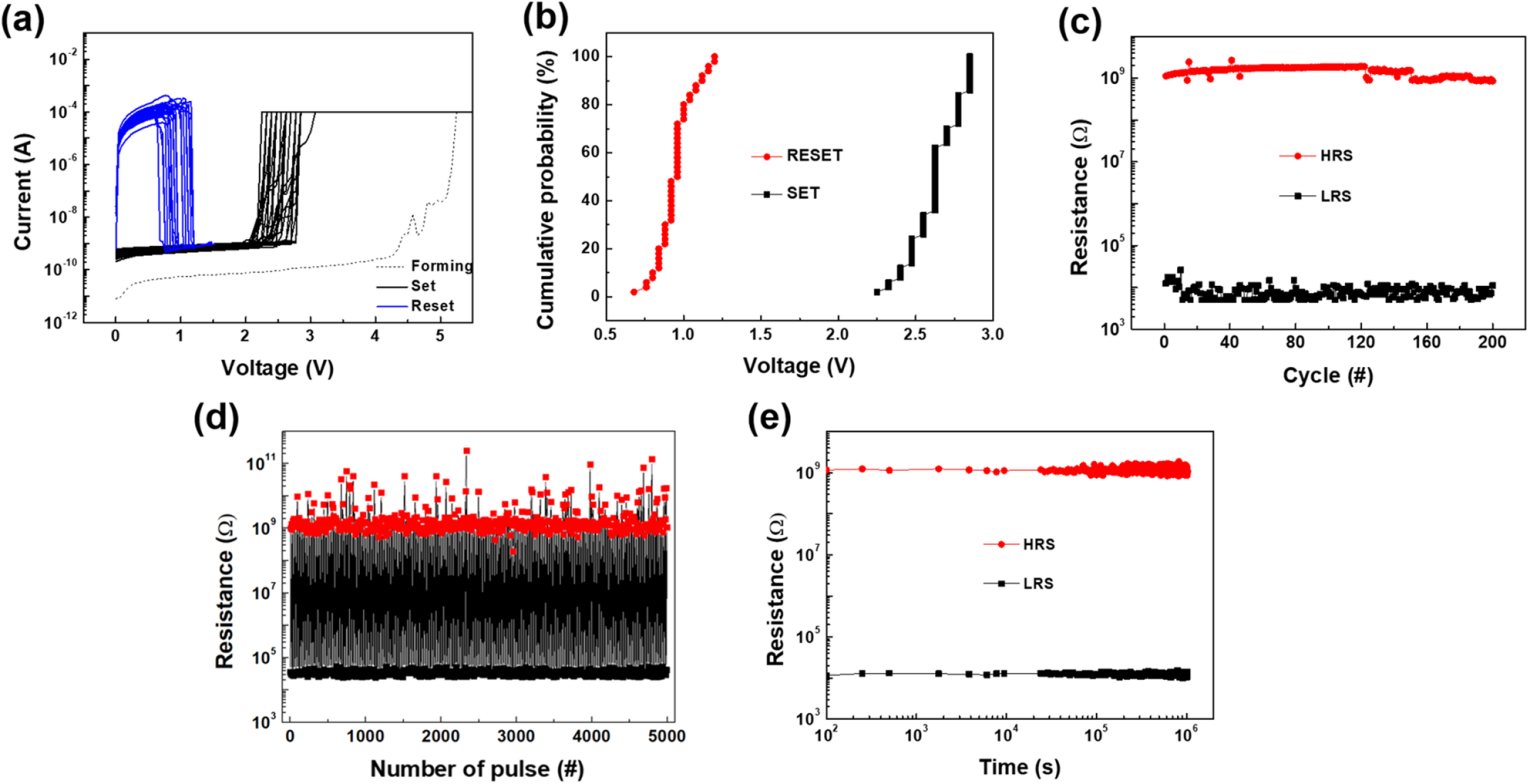
(**a**) Resistance switching behavior of the composite ReRAM embedded 1 wt% of the ST-CSWs. (**b**) Cumulative probability graphs of the SET and RESET voltages of the composite ReRAM device. (**c**) Endurance of the composite ReRAM device. (**d**) Result of the pulse switching test for the composite-based memory device with 1 wt% of ST-CSWs for 5,000 cycles. The SET and RESET pulse heights [duration] of 3.75 V [0.25 μs] and 1.65 V [0.25 μs], respectively. (**e**) Retention result of the composite ReRAM device at 358 K.

**Figure 4:**
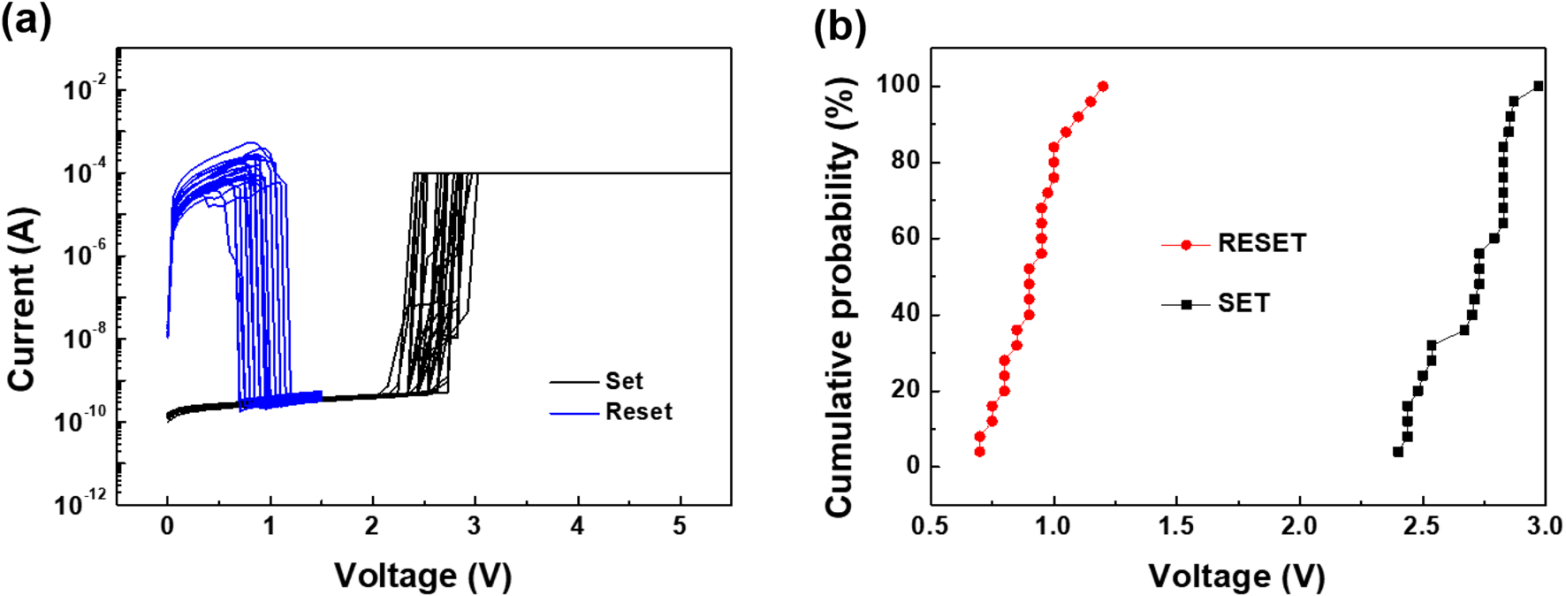
(**a**) *I–V* characteristics on 25 composite-based ReRAM cells with 1 wt% of ST-CSWs. The SET and RESET curves were collected at the 100th sweep cycle of each cell, respectively. (**b**) Cumulative probability of SET and RESET operating voltages on the cell-to-cell test.

We prepared the composite-based ReRAM device with the 300 nm-thick composite layers embedding 1 wt% of ST-CSWs in PVA polymer supporter (Supplementary Figure S1). Then, the *I-V* measurements were conducted for the device. The electroforming behavior was observed at 5.25 V, as shown in Fig. 3a. The forming process exhibited a two-step forming characteristic because the actual structure of the device has two metal–insulator-metal structures of Pt top electrode (TE)/TiO_2_/SWCNT and SWCNT/TiO_2_/Pt bottom electrode (BE). When the subsequent *I-V* sweep measurements were conducted up to 200 cycles, the resistance switching behavior showed the typical unipolar resistance switching (URS) behavior. The SET [i.e. resistance switching from a high resistance state (HRS) to a low resistance state (LRS)] and RESET behaviors (switching back from LRS to HRS) were operated successfully without any failure or degradation of the operation parameters (SET/RESET voltages and R_ON_/R_OFF_ ratio). The average values of the SET and RESET voltages were 2.63 ± 0.16 and 0.95 ± 0.11 V, respectively, demonstrating remarkably uniform switching operation within narrow voltage ranges. The distribution of the SET and RESET voltages, and LRS and HRS values for repeated 200 *I–V* cycles were summarized in Fig. 3b and 3c, respectively. As shown in Fig. 3d, the composite-based ReRAM device exhibited the reproducible switching behavior in a pulse switching test up to ~ 5,000 switching cycles. The R_ON_/R_OFF_ ratio during the test was about 10^4^. In addition, the resistance values and their standard deviations of the LRS and HRS were (3.48 ± 0.07) × 10^4^ and (8.1 ± 4.9) × 10^8^ Ω, respectively. This result obviously proved the stable switching operation of our memory device. When a retention test on the device was conducted at 358 K, the two resistance values were stable up to 10^6^ s (Fig. 3e). Furthermore, cell-to-cell tests for 25 cells were performed. Figure 4 and Supplementary Figure S3 show *I–V* curves and SET and RESET voltage values collected at the 100^th^ sweep cycle of each cell, respectively, exhibiting very uniform switching characteristics with narrow distribution (SET and RESET voltage of 2.69 ± 0.17 and 0.92 ± 0.13 V, respectively). This remarkable resistance switching operation is due to a confined electric field effect derived from unique structural contact between one-dimensional ST-CSW and two-dimensional electrode^32^. It is well-known that manipulating electric field in resistive switching memory device can control the nucleation and growth of conductive filament, achieving reliable resistive switching performance^3,33^. In addition, when we prepared the composite-based ReRAM device including 3 and 5 wt% of ST-CSWs, the resistance switching characteristics including pristine current level and forming voltage was similar to that of the composite-based ReRAM device with 1 wt% of ST-CSWs (Supplementary Figure S2). This result means that the 1 wt% of ST-CWSs in the PVA matrix is sufficient to connect between the top and bottom electrodes.

**Figure 5:**
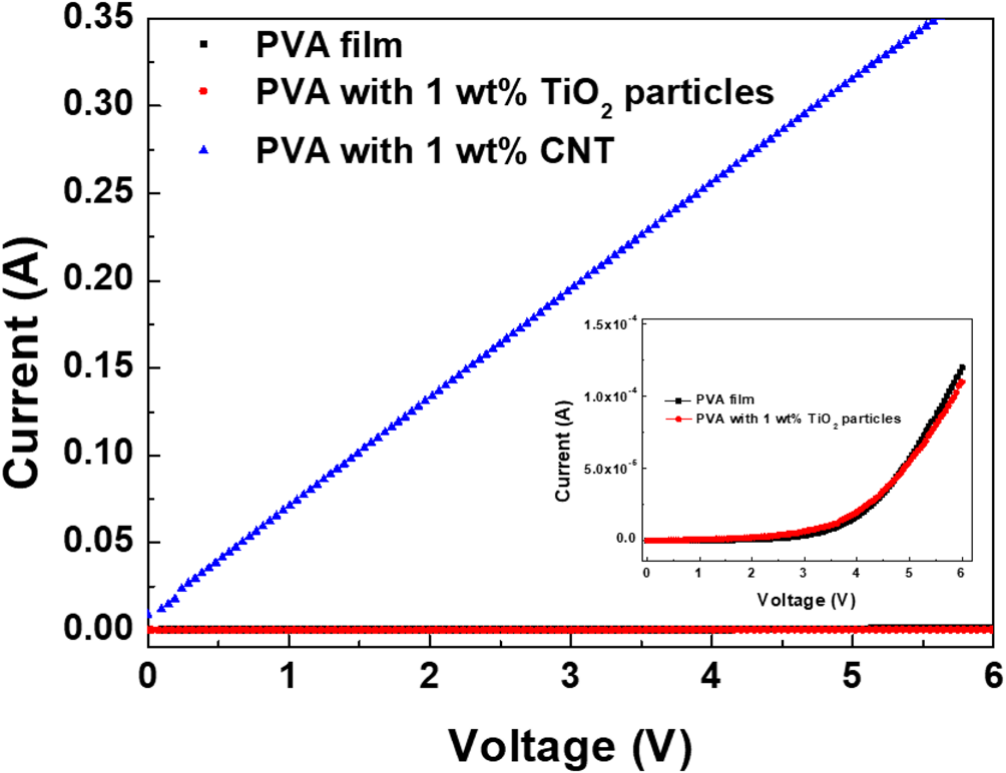
*I–V* curves of PVA film, PVA with 1 wt% TiO_2_ particles prepared by sol–gel process, and PVA with 1 wt% SWCNT. (inset) magnified *I–V* curves of PVA film, and PVA with 1 wt% TiO_2_ particles.

To demonstrate the effect of the ST-CSW on the meaningful resistance switching results, the neat PVA film, the composite film embedded 10 wt% of TiO_2_ particles prepared by the same sol–gel method with preparing ST-CSW, and the composite film with 1 wt% of SWCNT were examined (Fig. 5). The neat PVA film and the composite film introducing TiO_2_ particles exhibited the typical *I–V* curve of insulator when the voltages were applied up to 6 V. The composite film with 1 wt% of SWCNT showed the typical *I–V* curve of the conductor. Therefore, it can be concluded that the resistance switching behavior of the composite-based ReRAM device is originated from the CT-CSW.

**Figure 6:**
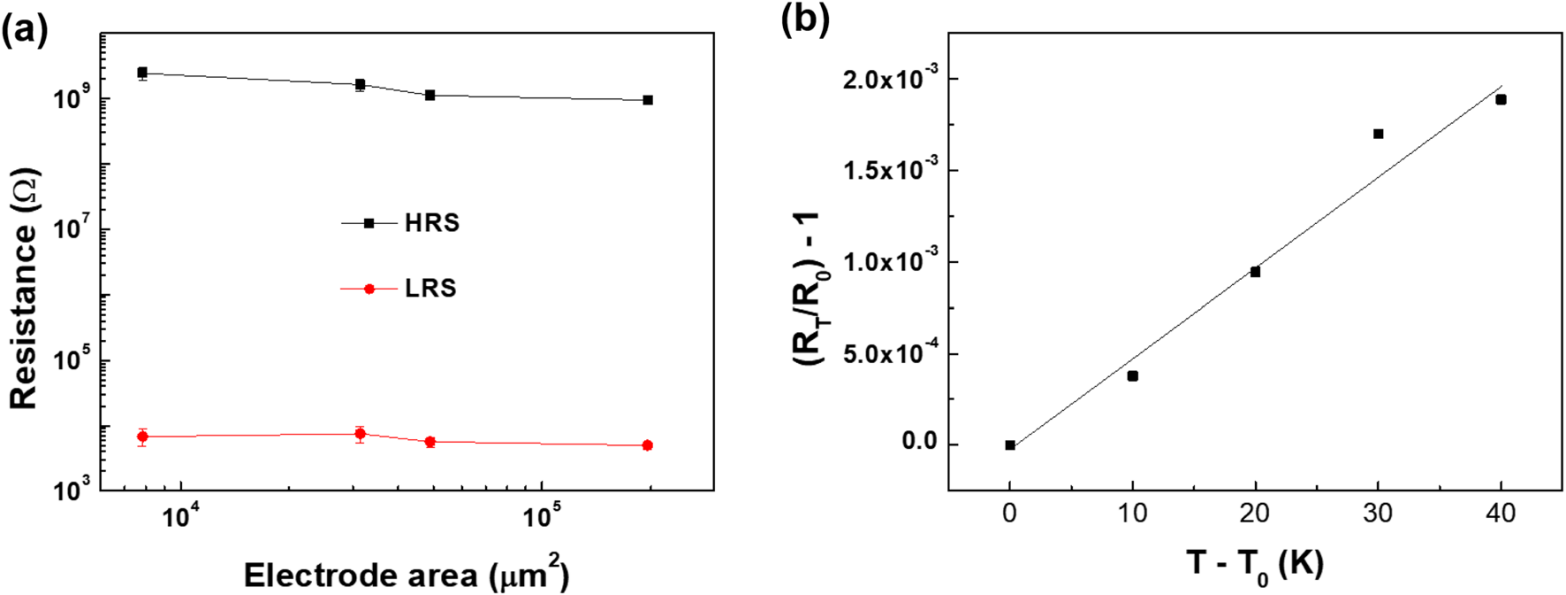
(**a**) Electrode-area dependent behaviors of the composite ReRAM device. (**b**) Temperature coefficient of resistance graph of the composite ReRAM device.

To identify the switching mechanism of the device, the area-dependent resistance switching behavior was examined because TiO_2_ is well-known as a material capable of both resistance switching mechanisms by the charge trapping or filament formation. The HRS and LRS values were independent of the varied TE sizes (Fig. 6a), implying that the resistance switching behavior of the device is originated from the filamentary conduction switching mechanism. To clarify this behavior, the temperature coefficient of resistance (TCR) test on the device was conducted with a temperature range from 303 to 343 K, based on the following equation:

1 $$R_{T} = R_{o} [1 + \alpha (T - T_{o} )],$$

where $\alpha$ is the TCR value. *R*_*o*_ and *R*_*T*_ are the resistance of the LRS at the initial temperature *T*_*o*_, and the resistance of the LRS at temperature *T*, respectively. The calculated $\alpha$ value from Fig. 6b of the device was 5.09 × 10^–5^ K^−1^. The value is comparable with the reported value of the Magnéli phase (1.47 × 10^–5^ K^−1^)^34,35^, implying that the resistance switching behavior of the device would be attributed to the formation of the conductive filament consisting of Magnéli phase by the electrical stimulus.

**Figure 7:**
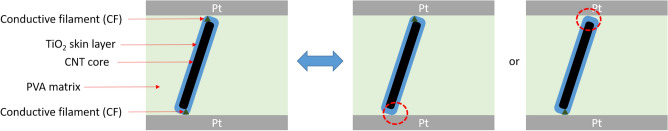
Schematic illustration of resistance switching behavior in the composite-based ReRAM device. The resistance switching behavior occurs at the contact region between Pt electrode and the TiO_2_ skin layer of the SWCNT@TiO_2_ core–shell nanowire.

On the basis of the above results, the proposed resistance switching mechanism is illustrated in Fig. 7. When the positive bias is applied to the Pt TE, the resistance of the TiO_2_ junction between the Pt TE and the SWCNT drops due to the phase transition from TiO_2_ to Ti_4_O_7_ in the TiO_2_ skin layer^36^. And then, the second filament will be subsequently formed in the same manner at the TiO_2_ junction between the SWCNT and Pt BE. The resistance state can be switched back LRS to HRS by a rupture of a formed conductive filament when a negative bias is applied to the Pt TE by joule heating effect derived from a higher current than the set compliance current.

**Figure 8:**
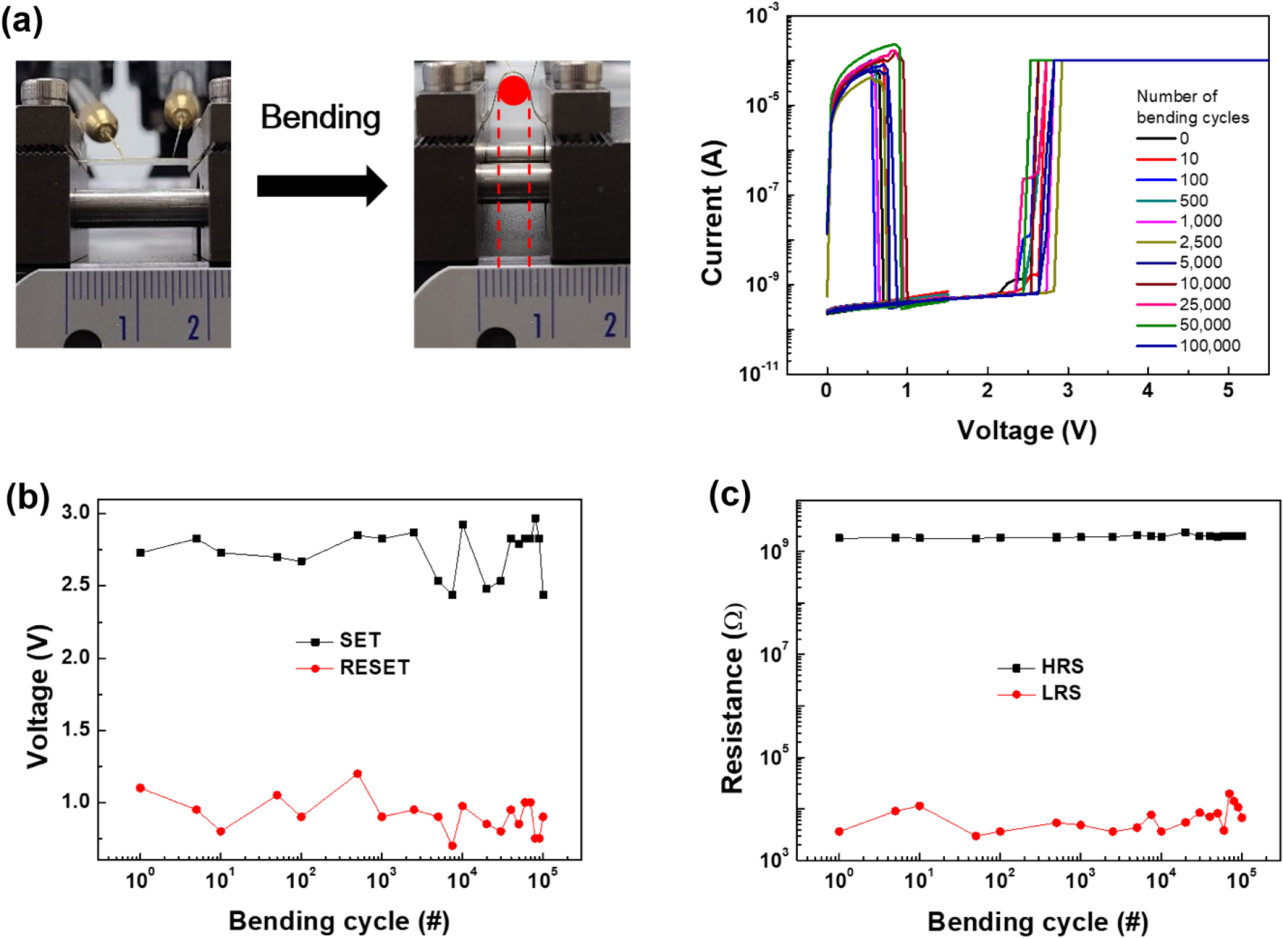
(**a**) Photograph of the bending test and *I–V* curves of the composite-type ReRAM device on the flexible test for 10^5^ cycles. (**b**) the distribution of SET/RESET voltages and (**c**) HRS/LRS trends during the bending test.

Finally, the feasibility of the composite-based ReRAM as a flexible memory device was examined, as shown in Fig. 8. For the flexible test, the composite film with ST-CSWs of 1 wt% was cast on poly(ethylene terephthalate) (PET) substrate with crossbar-patterned TE and BE (Supplementary Figure S4). During the flexibility examination, the device was deformed from a flat state to a bending radius of 2 mm, and the test was conducted up to 10^5^ bending cycles. The operating parameters (SET and RESET voltages and R_ON_/R_OFF_ ratio values) were maintained without any degradation. This remarkable stability against mechanical stress is related to the inherent elastic properties of the PVA matrix and the one-dimensional structure of the ST-CSW. Moreover, the physical interaction such as a hydrogen bonding between the TiO_2_ surface of the ST-CSW and the PVA matrix can be formed^37^.

## Conclusions

SWCNT@TiO_2_ core–shell wire was prepared as a novel resistance switchable filler in the nanocomposite resistance switching layer. The TiO_2_ skin layer on the surface of the SWCNT was uniformly formed by π–π interactions between the aromatic SWCNT surface and benzyl ring of BA, leading the uniform hydrolysis with the titanium precursor. And then, its resistance switching performance was investigated against mechanical and electrical noise. The nanocomposite resistance switchable devices showed notable resistance switching behavior with remarkably narrow operation parameters with a large R_ON_/R_OFF_ ratio. The reliable operation of our device was also sustained even in cell-to-cell tests for 25 cells. In addition, the notable resistance switching behavior with narrow operation parameters and the large R_ON_/R_OFF_ ratio was maintained against 10^5^ bending cycles without any deterioration and failure. Based on the above results, we conclude that the nanocomposite resistance switching layer based on one-dimensional SWCNT@TiO_2_ core–shell wire would be a promising platform for flexible electronics as an essential data storage component.

## Methods

0.5 wt% of Sodium dodecylbenzene sulfonate (SDBS) and 1 wt% of SWCNT (E.C. 2.0, Meijo Nano carbon Co.) was added in 50 mL of EtOH, and then the solution was tip-sonicated for 1 h (750 W and 40% amplitude condition). Benzyl alcohol (BA) and deionized water were added and mixed by magnetic stirring at 0 °C for 12 h. The tetrabutyl-orthotitanate (TBOT) was dissolved in ethanol and slowly dropped into the suspension. The final molar ratio of the mixture of TBOT:BA:EtOH:H_2_O was 0.25:50:x:5 (x = 10 and 30). After one hour reaction, the precipitates were purified by vacuum filtering with EtOH washing. The resulting particles were freeze-dried for 36 h. Finally, the particles were heat-treated (150 °C for 1 h followed by 400 °C for 1 h) in a tube furnace for the crystallization of the TiO_2_ skin layer. Pt bottom electrode (50-nm-thick) deposited on TiO_2_/SiO_2_/Si substrate of 1.25 × 1.25 cm was cleaned with acetone, deionized water, and then ethanol in an ultrasonic bath for 15 min, respectively. 1 wt% of ST-CSWs were mixed with 10 wt% Polyvinyl alcohol (PVA) solution (M_W_ ≈ 50,000 g/mol) in deionized water. And then, the mixture was stirred at 70 °C for 6 h. The solution containing ST-CSWs was spin-cast on the Pt BE/TiO_2_/SiO_2_/Si substrate, followed by solvent removal at 70 °C for 24 h. Finally, 50 nm-thick Pt top electrode (TE) with a dot shape was deposited via the sputtering method with a metal shadow mask whose hole diameters were varied of 100, 200, 250, and 500 μm, respectively. The morphological analyses of the pristine SWCNT, the ST-CSW, and the composite film were conducted by scanning electron microscopy (SEM, JSM-6701F, JEOL). The crystal structure of the ST-CSW was characterized by X-ray diffraction (XRD, Dmax 2500). The current–voltage (*I–V*) measurement was performed using an Agilent 4156C analyzer under ambient conditions. The pulse measurement was conducted using a semiconductor parameter analyzer, Keithley 4200-SCS, with Keithley 4225-PMU (pulse measurement unit).

## Supplementary information

Supplementary Information
