# Supplementary material for: Sustainable resistance switching performance from composite-type ReRAM device based on carbon Nanotube@Titania core–shell wires
Source: Sci Rep. 2020 Nov 2;10:18830. doi: 10.1038/s41598-020-75944-3 (PMC7608622; doi:10.1038/s41598-020-75944-3)
Supplement: Supplementary file 1 — Supplementary Information [file 41598_2020_75944_MOESM1_ESM.docx]

Supporting Information

Sustainable Resistance Switching Performance from Composite-type ReRAM Device based on Carbon Nanotube@Titania Core-Shell Wires

Youngjin Kim,^1,2,3^ Minsung Kim,^3,4^ Ji Hyeon Hwang,^3,5^ Tae Whan Kim,^2^ Sang-Soo Lee,^3,^* and Woojin Jeon^5,^*

^1^ The Research Institute of Industrial Science, Hanyang University, Seoul, 04763, Republic of Korea

^2^ Department of Electronic and Computer Engineering, Hanyang University, Seoul, 04763, Republic of Korea

^3^ Photo-Electronic Hybrids Research Center, Korea Institute of Science and Technology, Seoul, 02792, Korea

^4^ Department of Chemical and Biological Engineering, Korea University, Seoul 02841, Korea

^5^ Department of Advanced Materials Engineering for Information and Electronics, Kyung Hee University, Yongin 17104, Korea

*E-mail (W. Jeon): [woojin.jeon@khu.ac.kr](mailto:woojin.jeon@khu.ac.kr)

(S-S. Lee): [s-slee@kist.re.kr](mailto:s-slee@kist.re.kr)

**
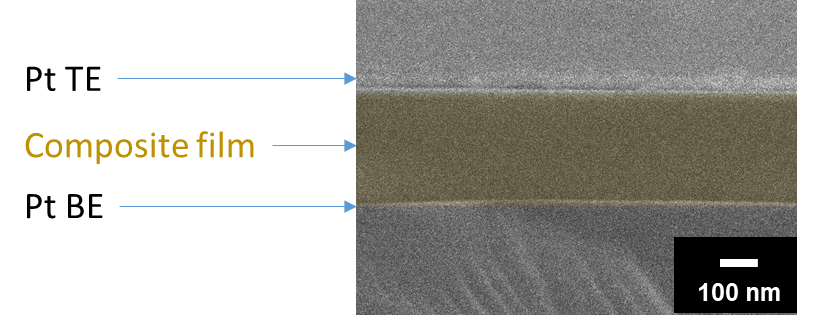
**

**Figure S1.** Cross-sectional SEM image of the composite-based ReRAM device.

**Figure S2.** *I-V* characteristics of the composite-based ReRAM devices with different concentrations of ST-SCWs.


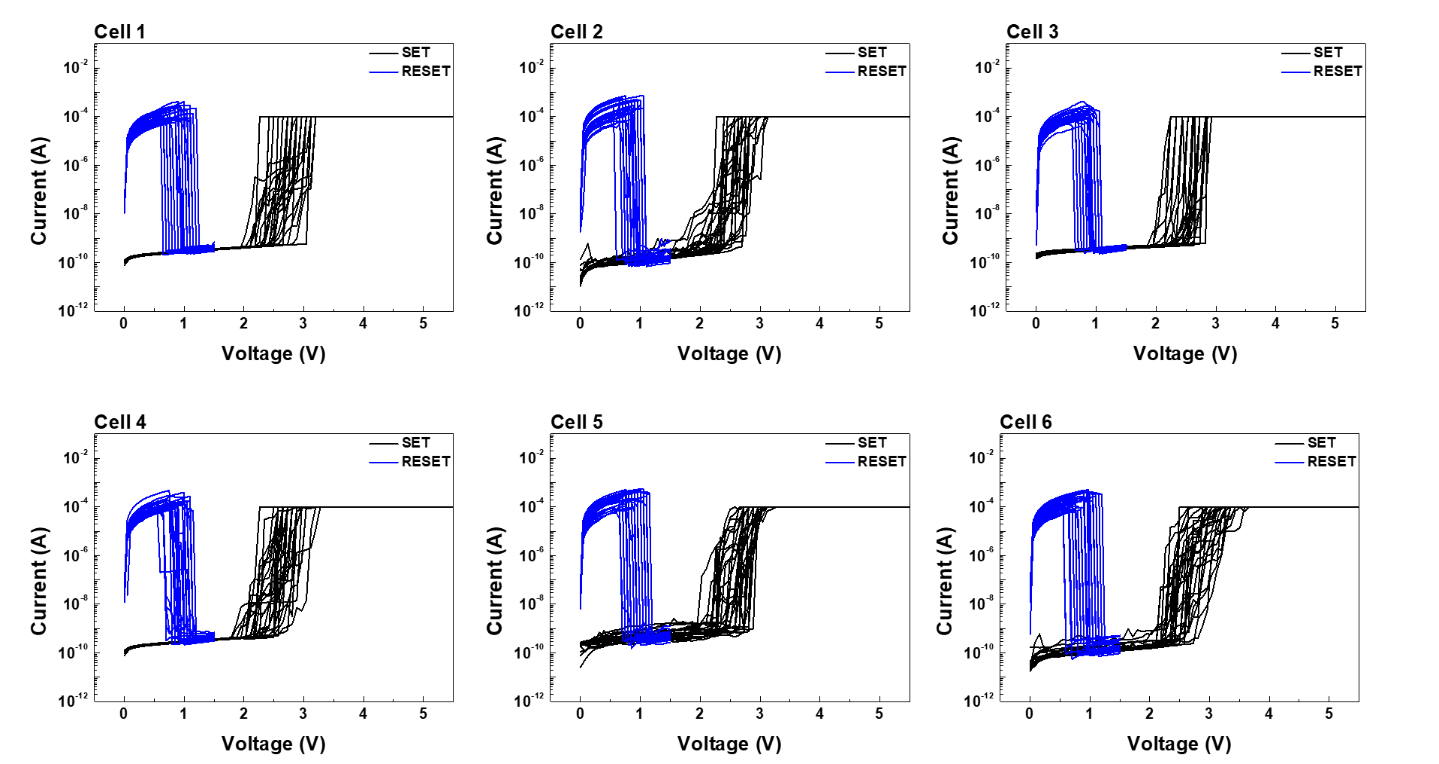


**Figure S3**. Consecutive *I-V* sweep results on six composite-based cells.

**
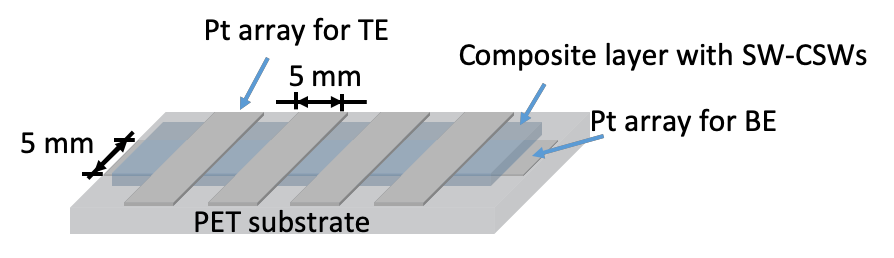
**

**Figure S4.** Schematic illustration of the flexible-type composite ReRAM device.
